# Comparison of axial length, anterior chamber depth and intraocular lens power between IOLMaster and ultrasound in normal, long and short eyes

**DOI:** 10.1371/journal.pone.0194273

**Published:** 2018-03-15

**Authors:** Jing Dong, Yaqin Zhang, Haining Zhang, Zhijie Jia, Suhua Zhang, Xiaogang Wang

**Affiliations:** 1 Department of Ophthalmology, The First Hospital of Shanxi Medical University, Shanxi, P.R. China; 2 Shanxi Eye Hospital, Shanxi, P.R. China; University of California Berkeley, UNITED STATES

## Abstract

**Purpose:**

To compare the axial length (AL), anterior chamber depth (ACD) and intraocular lens power (IOLP) of IOLMaster and Ultrasound in normal, long and short eyes.

**Methods:**

Seventy-four normal eyes (≥ 22 mm and ≤ 25 mm), 74 long eyes (> 25 mm) and 78 short eyes (< 22 mm) underwent AL and ACD measurements with both devices in the order of IOLMaster followed by Ultrasound. The IOLP were calculated using a free online LADAS IOL formula calculator.

**Results:**

The difference in AL and IOLP between IOLMaster and Ultrasound was statistically significant when all three groups were combined. The difference in ACD between IOLMaster and Ultrasound was statistically significant in the normal group (P<0.001) and short eye group (P<0.001) but not the long eye group (P = 0.465). For the IOLP difference between IOLMaster and Ultrasound in the normal group, the percentage of IOLP differences <|0.5|D, ≥|0.5|D<|0.75|D, ≥|0.75|D<|1.0|D, and ≥|1.0|D were 90.5%, 8.1%, 1.4% and 0%, respectively. For the long eye group, they were 90.5%, 5.4%, 4.1% and 0%, respectively. For the short eye group, they were 61.5%, 23.1%, 10.3%, and 5.1%, respectively.

**Conclusions:**

IOLMaster and Ultrasound have statistically significant differences in AL measurements and IOLP (using LADAS formula) for normal, long eye and short eye. The two instruments agree regarding ACD measurements for the long eye group, but differ for the normal and short eye groups. Moreover, the high percentage of IOLP differences greater than |0.5|D in the short eye group is noteworthy.

## Introduction

It is well known that accurate axial length (AL), keratometric value and anterior chamber depth (ACD) measurements are of essential importance for intraocular lens power (IOLP) calculation. There are two common types of biometry based on different working principles. The first type is noncontact optical biometry, which is designed using partial coherence interferometry to provide ACD, AL and keratometry with a single measurement. The second type is contact ultrasound biometry using 10-MHz ultrasound waves to measure AL, ACD, and lens thickness [1].

The IOLMaster (Carl Zeiss Meditec, Germany), as a partial coherence interferometer, provides highly repeatable and reproducible corneal parameters, ACD and AL values [1]. It measures the optical path length from the corneal anterior surface to the retinal pigment epithelium as AL [2]. Moreover, it can provide different IOLP calculation formulas even for different IOL models, which is of great help when planning IOL implantation in the clinic [1]. However, ocular biometric parameters cannot be successfully captured for patients with subcapsular and dense cataracts. Therefore, ultrasound biometry cannot be replaced by optical biometry in all cases.

Ultrasound biometry can provide ACD, lens thickness, vitreous body length and AL (from the corneal vertex to the internal limiting membrane) and is performed by immersion of the ultrasound probe in a saline-filled shell or by applanation of the probe to the cornea after surface aneasthesia [2]. Generally, the immersion technique is considered much more accurate and provides longer measurements than the contact technique [3]. A recent study of 36 subjects with repeated measurements by both the contact and immersion techniques showed that they were comparable, with no clinically significant differences in the mean AL measurements [4].

The accurate calculation of IOLP is a critical factor for optimizing patients’ outcomes, especially for different AL eyes. Several modern mathematical formulas, such as Hoffer Q, Holladay I, Holladay I with Koch adjustment, Haigis, and SRK/T, have been used in the clinic to improve the accuracy under some specific conditions. A recent study showed that an IOL super formula (LADAS formula) is capable of providing the most accurate calculations and determining the ideal IOLP calculation for an individual eye under all situations [5]. Most of previous research about IOLMaster and Ultrasound were small sample size studies with no AL sub-group comparisons [2]. Based on clinical surgeons experience, IOLP calculation formulas choice were commonly divided by the AL boundary of 22 mm and 25 mm. We make the hypothesis that different AL sub-groups may influence the measurement consistency and IOLP between them. Based on the assumption that the same individual optical ACD and K readings from the IOLMaster were used for each eye’s IOLP calculation, we only want to check whether the potential AL measurement difference resulted in a clinically significant IOLP difference. Therefore, the purpose of this study was to investigate the AL, ACD, and IOLP (using LADAS formula) differences with IOLMaster and Ultrasound in normal, short and long eyes with a relatively large sample size.

## Materials and methods

This study was performed at Shanxi Eye Hospital between February, 2017 to June, 2017 and only pre-operation cataract eyes were included in this study. The research protocols were approved by the institutional review boards of Shanxi Eye Hospital and carried out in accordance with the tenets of the Declaration of Helsinki. Written informed consent was obtained from each subject after he or she was given an explanation of the nature of the study.

### Subjects

We chose to study only Han Chinese subjects to eliminate the possible influences of different ethnic groups. The inclusion criteria for the studied group included: a best-corrected visual acuity (BCVA) less than 8/20, normal slit-lamp and fundoscopy examinations, an intraocular pressure (IOP) < 21 mmHg, central fixation sufficiently stable to perform image capture and no history of ocular or systemic corticosteroid use. Subjects with keratoconus, previous corneal lesions and prior surgery in the cornea, glaucoma or posterior abnormalities, such as choroidal neovascularization, retinoschisis, retinal detachment or macular holes, and those with missing data (failed to cooperate with IOLMaster or Ultrasound examination, severe cataracts failed IOLMaster examination, IOLMaster signal-to-noise ratio less than 2.0) were excluded. Eyes were divided into 3 sub-groups based on IOLMaster AL values: normal group (AL ≥ 22 mm and ≤ 25 mm), short eye group (AL less than 22 mm), and long eye group (AL more than 25 mm).

### Data acquisition

The ACD and AL were measured via IOLMaster then Ultrasound with no pupil dilation. Each measurement was repeated ten times in each eye, and the averaged value was used in the analysis. The software was version 7.5 for IOLMaster. The subject was asked to place his chin on the chin rest and press his forehead against the forehead strap. The subject’s eye was aligned to the visual axis by a central fixation light or target. A single trained operator performed all of the examinations using both devices. The keratometry index was 1.3375, and the ACD value was the distance from the corneal epithelium to the anterior lens surface. All 10 readings with a signal-to-noise ratio greater than 2.0 were acceptable for final data analysis (individual participants’ data are presented in S1 Dataset).

A handheld A-scan ultrasound biometry device (ECHOGRAPH–Model: AXIS–II PR, QUANTEL MEDICAL, FRANCE) with a 10-MHz A-scan biometry probe was used for the contact AL measurements. Ultrasound velocities of 1532 millisecond^-1^ for the anterior chamber and vitreous and 1641 milliseconds^-1^ for the lens were used. One drop of topical anesthetic (0.4% oxybuprocaine hydrochloride eye drops) was instilled into the eye 3 minutes before ultrasound biometry was performed. For each device, a single trained operator performed all of the examinations.

### Intraocular lens power calculation

The free online LADAS super formula (http://www.iolcalc.com/) was used for IOLP calculation in each group (version 1.0b). We assume that each eye would use the same A constant (118.0), K index (1.3375), IOLMaster individual optical ACD and keratometric readings to observe the potential effect of AL measurement on IOLP calculation. Moreover, we set the target refraction to zero for all the IOLP calculations.

### Statistical analysis

Statistical analysis was performed with SPSS ver. 13.0. The statistical significance of the inter-device differences in ACD, AL and IOLP was evaluated with the paired two-tailed t-test. The inter-device agreement was evaluated using Bland-Altman analysis. The inter-device differences were plotted against their means, and the 95% limits of agreement (LoA) were determined using this method. The significance level for all of the tests was set at 5%.

## Results

According to IOLMaster AL values, a total of 74 eyes (≥ 22 mm and ≤ 25 mm), 74 eyes (> 25 mm) and 78 eyes (< 22 mm) were included in the normal group, long eye group and short eye group, respectively (Table 1).

**Table 1:** Characteristics of the study groups.

	Normal Group	Long Eye Group	Short Eye Group
Patients, n	74	74	78
Eyes, n	74	74	78
Age (yrs)	71 ± 13	60 ± 12	67 ± 10
Flat K (D, by IOLMaster)	44.21 ± 1.40	43.84 ± 1.77	45.52 ± 1.57
Steep K (D, by IOLMaster)	45.21 ± 1.58	44.99 ± 1.93	46.65 ± 1.51

D = Diopter; K = keratometry.

The difference in AL and IOLP between IOLMaster and Ultrasound was statistically significant for the combination of all three groups. The difference in ACD between IOLMaster and Ultrasound was statistically significant in the normal group and short eye group but not in the long eye group (Table 2).

**Table 2:** Axial length, anterior chamber depth and intraocular lens power data comparison between IOLMaster and Ultrasound in each group.

	IOLMaster	Ultrasound	I—U	P*
**Normal Group (n = 74)**				
AL (mm)	23.09 ± 0.72	23.14 ± 0.71	-0.05 ± 0.08	<0.001
ACD (mm)	2.84± 0.46	3.02 ± 0.46	-0.18 ± 0.23	<0.001
IOLP (D)	20.12 ± 2.24	19.96 ± 2.26	0.17 ± 0.26	<0.001
**Long Eye Group (n = 74)**				
AL (mm)	28.27 ± 2.57	28.23 ± 2.52	0.04 ± 0.12	0.006
ACD (mm)	3.54 ± 0.37	3.56 ± 0.42	-0.02 ± 0.24	0.465
IOLP (D)	6.37 ± 6.51	6.45 ± 6.42	-0.08 ± 0.29	0.015
**Short Eye Group (n = 78)**				
AL (mm)	21.44 ± 0.50	21.54 ± 0.50	-0.10 ± 0.08	<0.001
ACD (mm)	2.42 ± 0.34	2.56 ± 0.39	-0.14 ± 0.21	<0.001
IOLP (D)	24.40 ± 2.51	23.97 ± 2.42	0.42 ± 0.32	<0.001

ACD = anterior chamber depth; AL = axial length; IOLP = intraocular lens power; I = IOLMaster; n = number of eye; U = ultrasound.

Note

*Paired two-tailed t-test; Values were displayed as mean ± standard deviation.

**Fig 1:**
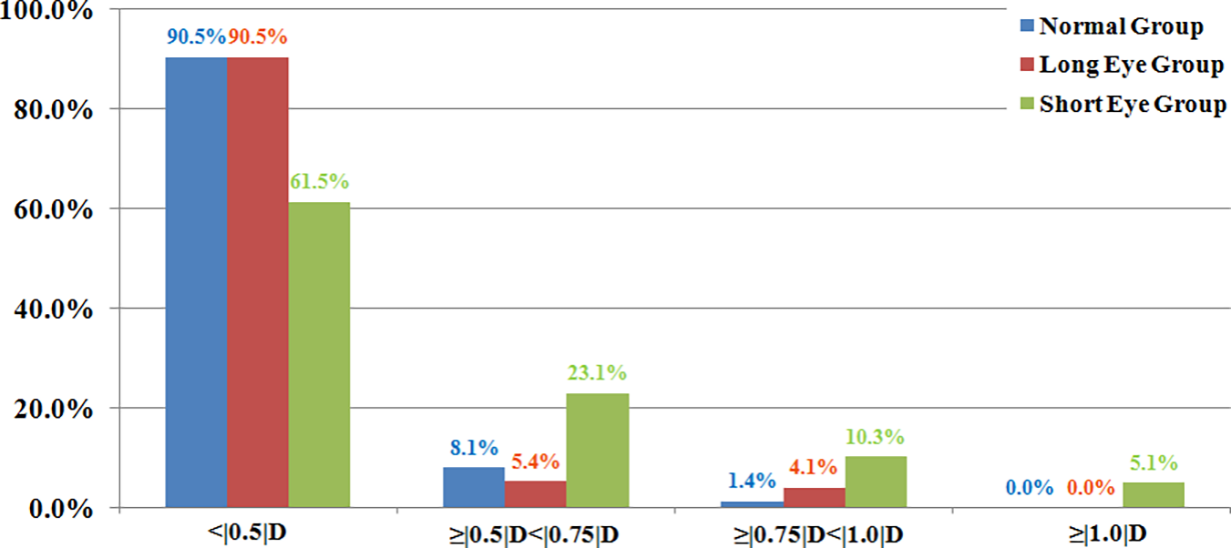
Intraocular lens power difference of different ranges between IOLMaster and Ultrasound in each group.

For the IOLP difference between IOLMaster and Ultrasound in the normal group, the percentages of IOLP differences <|0.5|D, ≥|0.5|D<|0.75|D, ≥|0.75|D<|1.0|D, and ≥|1.0|D was 90.5% (67/74), 8.1% (6/74), 1.4% (1/74) and 0% (0/74), respectively. For the long eye group, they were 90.5% (67/74), 5.4% (4/74), 4.1% (3/74) and 0% (0/74), respectively. For the short eye group, they were 61.5% (48/78), 23.1% (18/78), 10.3% (8/78), and 5.1% (4/78), respectively (Fig 1). The percentages of subjects with IOLP in different ranges (IOLMaster—Ultrasound) are summarized in Table 3.

**Table 3:** Percentages and case numbers of intraocular lens power in different ranges of the two devices in each group.

	IOLP (I—U)			
	-1.0 D ~ 0 D	0 D	D ~1.0 D	≥1.0 D
**Normal Group**	24.3% (18/74)	2.7% (2/74)	73.0% (54/74)	0% (0/74)
**Long Eye Group**	56.8% (42/74)	6.8% (5/74)	36.4% (27/74)	0% (0/74)
**Short Eye Group**	7.7% (6/78)	1.3% (1/78)	85.9% (67/78)	5.1% (4/78)

D = diopter; IOLP = intraocular lens power; I = IOLMaster; U = ultrasound

**Fig 2:**
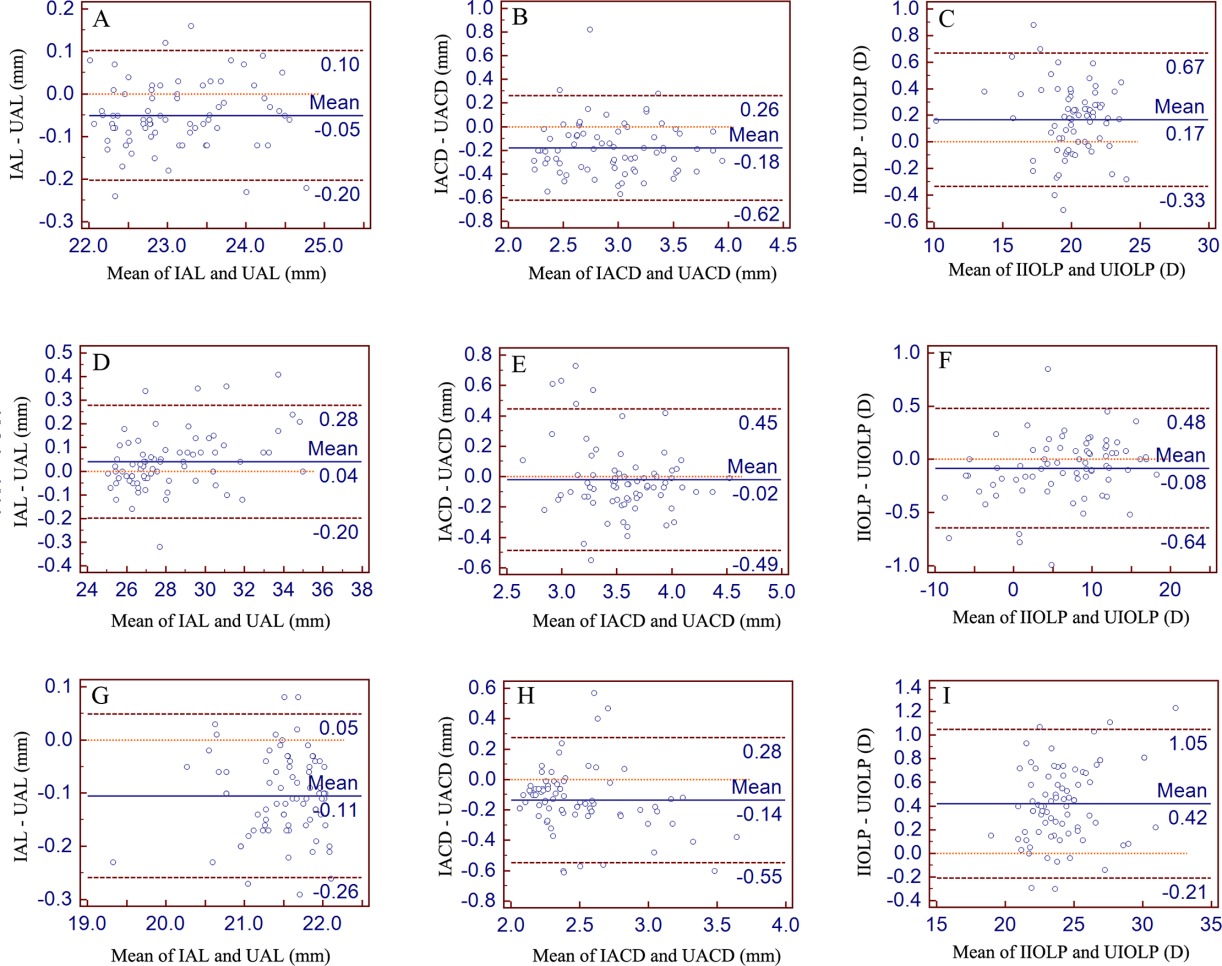
Bland-Altman plots for the axial length (AL), anterior chamber depth (ACD), and intraocular lens power (IOLP) comparing the IOLMaster (I) with the Ultrasound (U). In each panel, the 95% limits of agreement (LoA) mean difference, lower and upper limits were demonstrated as blue solid line and brown dotted line, respectively. Moreover, the corresponding values of LoA mean differnence, lower and upper limits were also demonstrated in each panel. Panel A, B, and C for the normal group, Panel D, E, and F for the long eye group and Panel G, H, and I for the short eye group.

For the normal group, the width of the LoA intervals for the AL, ACD, and IOLP were 0.30 mm, 0.88 mm, and 1.0 D, respectively; for the long eye group, the width of the LoA intervals for the AL, ACD, and IOLP were 0.48 mm, 0.94 mm, and 1.12 D, respectively; for the short eye group, the width of the LoA intervals for the AL, ACD, and IOLP were 0.31 mm, 0.83 mm, and 1.26 D, respectively (Fig 2). Moreover, the LoA mean differences, lower and upper limits values for the AL, ACD, and IOLP in each group were displayed in Fig 2.

## Discussion

With the increasing demands concerning more exact postoperative refractive error and higher patient expectations, there has been more of a focus in the field of refractive cataract surgery, especially for long and short eyes, on accurate ocular biometry measurement and predictable IOLP calculation of the eye, such as AL, ACD, anterior and posterior keratometry, lens thickness, and all types of improved IOLP formulas. We compared optical and ultrasound biometry parameters, such as AL, ACD and IOLP, in normal, long and short eyes in this study. The results demonstrated that IOLMaster and Ultrasound have statistically significant differences in AL measurements and IOLP calculations for normal, long and short eyes. The two instruments agree on ACD measurements for the long eye group but differ in the normal and short eye groups. Moreover, a relatively higher percentage of IOLP differences greater than |0.5|D should be noticed in the short eye group.

Previous studies have shown that IOLMaster and contact Ultrasound have acceptable repeatability of AL and ACD measurements for the clinic [6–8]. Compared to IOLMaster, significantly higher AL values were observed via Ultrasound for the normal and short eye groups. Inversely, significantly lower AL values were found via Ultrasound in the long eye group. The AL differences between optical biometry and ultrasound biometry could mainly be explained by several potential reasons. Firstly, different imaging modalities have different resolutions. For ultrasound, the accuracy of AL measurement was approximately 0.12 mm, which is less than the 0.012 mm from the optical AL measurement [9]. Secondly, there is a different measurement boundary between IOLMaster and Ultrasound biometry. Compared to Ultrasound, IOLMaster should generally provide approximately 200 μm longer values, which is approximately the average retinal thickness [10]. However, IOLMaster demonstrated approximately 0.04 ± 0.12 mm higher AL values than Ultrasound in the long eye group but demonstrated approximately 0.05 ± 0.08 mm and 0.10 ± 0.08 mm lower AL values than Ultrasound in the normal and short eye groups in this study. This measurement inconsistency has been reported in some previous studies [9,11,12]. Thirdly, Ultrasound measures the anatomic axis of the eye, which is different from the optical AL along the visual axis (tilted approximately 5° horizontally and 1° vertically relative to the anatomic axis) [9,13].

The ACD measurements of the IOLMaster were higher than Ultrasound in each group, and a statistically significant differences were found in the normal and short eye group in this study (the width of the LoA intervals being 0.88 mm, 0.94 mm and 0.83 mm for the normal, long eye and short eye groups, respectively). This result was similar to that from the study by Elbaz et al.: Ultrasound measured a significantly higher ACD compared to IOLMaster (the width of the LoA interval being 0.65 mm) [14]. However, the result was the opposite of that from the study by Hashemi et al.: Ultrasound measured a significantly lower ACD compared to IOLMaster (the width of the LoA interval being 0.54 mm) [15]. The contradictory results may be attributed to: 1) the measurement method difference: Ultrasound biometry relies on ultrasound, while IOLMaster is based on partial coherence interferometry; 2) the potentially different accommodation states during each measurement [16]; and 3) the possibility that operator experience influences the measurement performance, especially for Ultrasound biometry [17].

A study by Ladas et al. demonstrated that the LADAS IOL super formula incorporates the ideal segments from each of the existing formulas, such as Hoffer Q, Holladay I, Holladay I with Koch adjustment, Haigis and SRK/T, and it uses the ideal IOL formula for each individual eye [5]. Therefore, we used the LADAS IOL super formula for IOLP calculation in our study. Using the LADAS formula, the IOLP was essentially comparable between the two devices for the normal and long eye groups. For the normal and long eye groups, the mean IOLP difference between the two devices was less than |0.5|D in approximately 90% of cases, and no case was more than |1.0|D. These findings indicate that the differences are clinically negligible for most subjects having cataract surgery with normal and long eye AL. Hence, the AL measurements with the two devices can be interchangeable using the same keratometry, optical ACD and A-constant for IOLP calculation for AL more than 22 mm. However, the mean IOLP difference less than |0.5|D between the two devices was 61.5%, and 5.1% of the eyes demonstrated an IOLP difference greater than |1.0|D for the short eye group. The outcomes indicate that the differences between the two devices are clinically noticeable for a large proportion of short eye cataract patients.

A few limitations of this study should be noted. First, only the LADAS IOL super formula was used for the IOLP calculation comparison between the two devices in each group, which should be considered when using the results. Therefore, we will compare more formulas in a future study. Second, this study included only Chinese subjects and therefore cannot be directly generalized to different ethnic backgrounds. Third, we performed all of the AL and ACD measurements on undilated pupils, which allowed the subjects to more easily fixate on the fixation target in the examination. However, without cycloplegia, the potential influences of accommodation on consecutive AL, ACD measurements cannot be excluded [18,19]. Despite these limitations, this prospective study to investigate the interchangeability of AL, ACD and IOLP between IOLMaster and Ultrasound in different AL eyes provides useful information for clinical practice.

In conclusion, The IOLMaster and Ultrasound have statistically significant differences in their AL measurements and in IOLP (using LADAS formula) for normal, long and short eyes. The difference between the two devices in the normal and long eye groups were clinically negligible. The two instruments agree on ACD measurement for the long eye group but differ in the normal and short eye groups. Moreover, the high percentage of IOLP differences more than |0.5|D in the short eye group should be noted.

## Supporting information

S1 Dataset Submission data reduction. (XLSX)
